# Supplementary figures and images for: Systematic Analysis of Cotton RING E3 Ubiquitin Ligase Genes Reveals Their Potential Involvement in Salt Stress Tolerance
Source: Int J Mol Sci. 2025 Jan 3;26(1):359. doi: 10.3390/ijms26010359 (PMC11720228; doi:10.3390/ijms26010359)

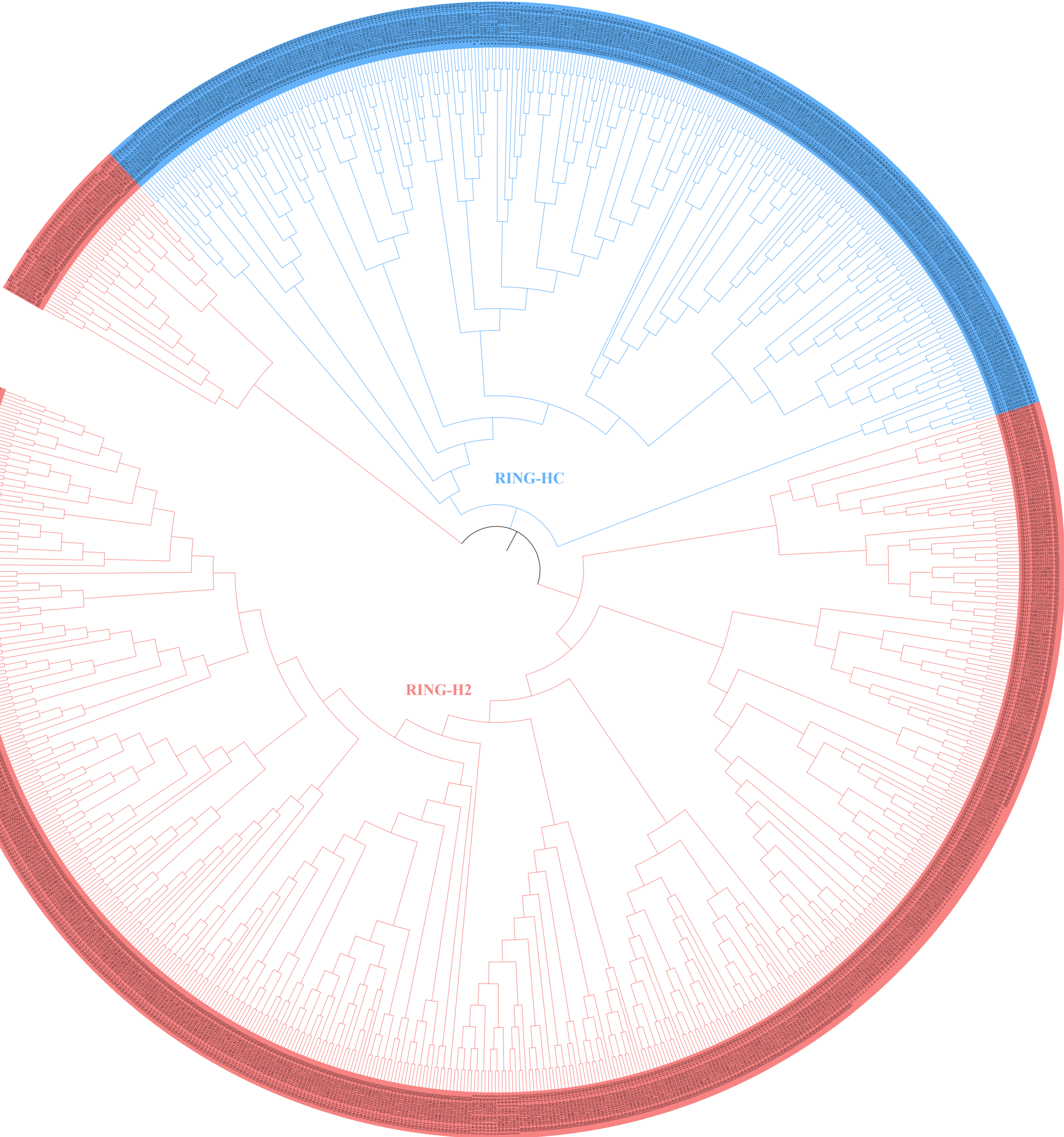

Supplement: Supplementary file 1 [file ijms-26-00359-s001.zip › Figure S1.pdf]

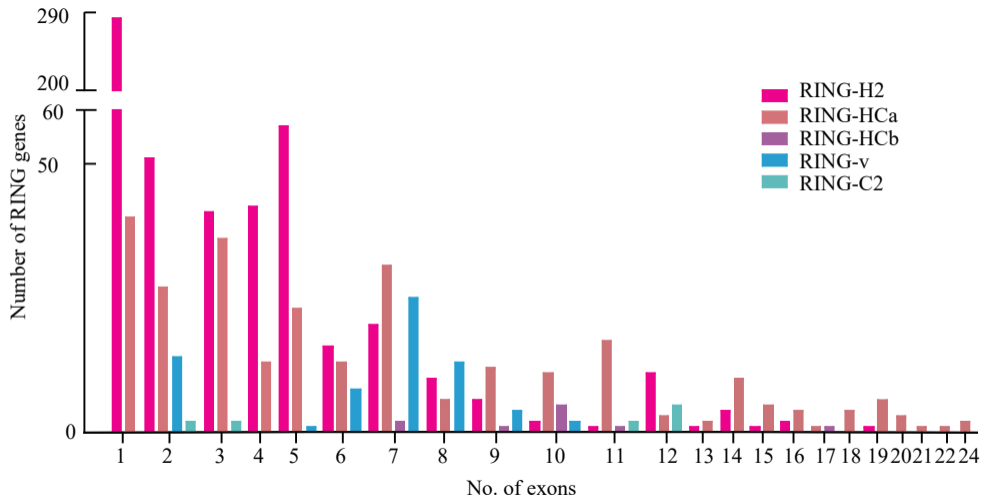

Supplement: Supplementary file 1 [file ijms-26-00359-s001.zip › Figure S2.pdf]

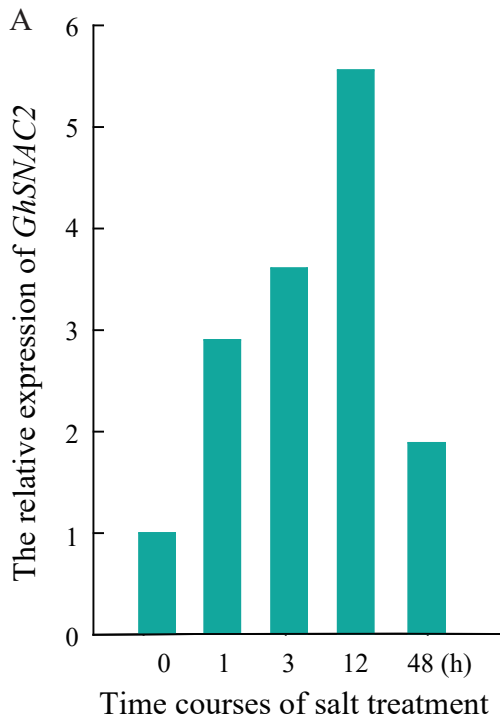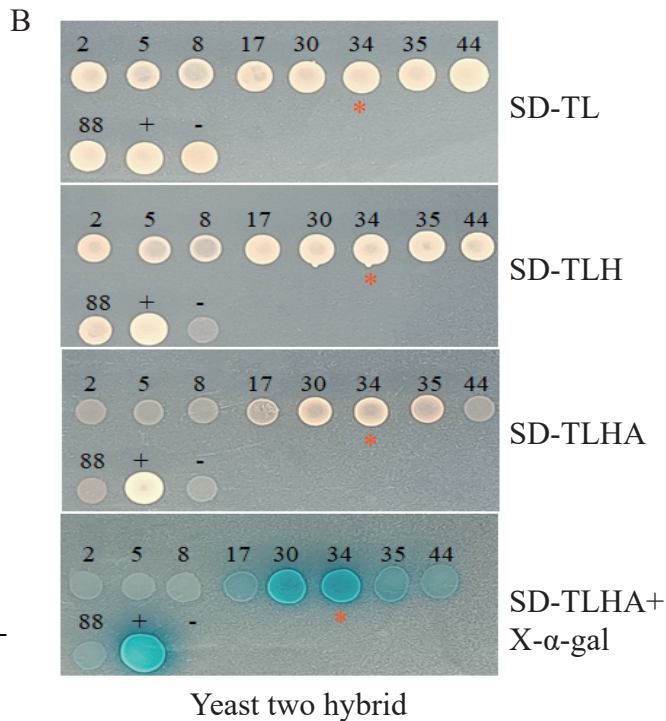

Supplement: Supplementary file 1 [file ijms-26-00359-s001.zip › Figure S3.pdf]
